# Scirrho-Contracted Rectum—Valvular Obstruction—With Comments

**Published:** 1829-01-01

**Authors:** 


### XXXII.

### SCIRRHO-CONTRACTED RECTUM—VALVULAR OBSTRUCTION.

The following melancholy case will disclose a source of obstruction to the evacuation of the fæcal matters which is far more common than is generally supposed. It will be best understood by a detail of the case.

Dr. De Leon (of Columbia) was called to Mrs. B. a lady, 25 years of age, on the 2d of March 1828, being then in her third pregnancy. For two months previously she had found much difficulty in evacuating her bowels—and for 30 days she had had no alvine evacuation at all! The urinary passage was also much obstructed, excepting in some particular postures of the body. Much purgative medicine had been prescribed, with no other effect than increasing the tenesmus, and therefore she had desisted, of late, from all medicines, except laudanum, for the relief of pain. Her general health did not appear affected—her appetite was good, but she restrained it. The doctor inferred, from this statement, that there was either a stricture in the intestine, or some tumour pressing on it.

"After examining the abdominal tumour externally, and finding it very voluminous and uniform, and not being able to trace the fundus of the uterus; making the necessary preparation, I placed her in a proper position, and passing my hand, well lubricated, into the vagina, it was soon opposed by a very firm body, which I traced to be the fundus of the uterus. That viscus must have been retroverted, by her account, at least nine or ten weeks before the period alluded to; as she then considered herself between the fourth and fifth months of utero-gestation, having felt her child, but observed at the same time that she had borne two children, but the movement she then experienced was very unlike that she felt in her former pregnancies. Many of the rational symptoms of pregnancy being absent, a very strong doubt was then created in my mind whether the uterus contained a child or some foreign body, the indication being the same in either case. I proceeded to restore the uterus to its natural situation, in which I succeeded, after a few patient trials. The vagina was greatly relaxed, owing to a profuse mucous discharge, which occurred about the period of her last catamenial discharge. Enjoining the most exact repose in a horizontal posture, she very soon passed urine freely.

"My next care was to examine the rectum, which I did by passing into it a flexible catheter of large size, armed with a syringe. But on attempting to pass the fluid out of the syringe into the bowel, I found it to be impossible. I made another trial with a wax bougie, with as little success. The patient being now much fatigued, I deferred any other mode of examination at that time, but prescribed a mild sudorific draught at bed time, and a gruel diet.

My next examination was made by the introduction of one finger into the vagina and another into the rectum. This examination instructed me conclusively, that the intestinal tube had undergone a change of structure; which fact was fully corroborated by the finger in the vagina, as far as it could reach. The capacity of the bowel below the stricture, was greatly enlarged; that above it, much narrowed, grasping the tip of the finger firmly as a hard ring. A soft membrane, which I concluded to be the mucous membrane of the bowel from above the seat of disease, protruded through this ring; not unlike what occurs frequently in protracted dysentery and diarrhœa. This membrane was very yielding, forming a kind of pouch, and moving readily before the finger. The nature of the case being clearly shown, I did not disguise from the husband her danger, and candidly prognosticated what I believed would be the issue. But leaning on 'hope' and her 'youth,' he could not persuade himself that the prospect was so unpromising.

"The paroxysms of pain were at this juncture severe, referred altogether to the epigastric region, attended with nausea and occasional vomiting. I prescribed such palliatives from time to time, as each indication seemed to me to require, and I used a flexible bougie of proper size for one week perseveringly. Finding not the smallest advantage gained, I next resorted to the tallow bougie with as little success, and lastly, to the metallic. Finding my efforts totally unavailing, and the accumulation in the bowels painfully increasing daily, and now producing violent spasm, I proposed a consultation with my intelligent friend, Dr. EDWARD FISHER, Sen. who politely attended. After carefully considering the case, he remarked to me that the bougie was the only course which promised any benefit under such unpromising circumstances, and advised a perseverance in its application, increasing their size as any advantage might be gained. This course was unremittingly pursued for two weeks longer without the smallest amendment that I could discover. In this truly distressing conjuncture, distressing beyond the power of description, from the insupportable accumulation in the bowels, her agony amounted to torture from this cause alone, superadded to which, she suffered under retention of urine.

"Perplexed, and perceiving no alternative for the relief of her agonizing sufferings, I again urged the necessity of another consultation, (Dr. Fisher being then absent.) Two gentlemen of standing in their profession were called in; they attended, and after detailing to them the whole progress of the disease, with the treatment, and making a manual examination in their presence, I proposed to divide the membrane, (before alluded to,) with a bistoury carefully introduced along the forefinger, on the ground that we should at least have an outlet for the fæces, which the membrane obstructed as a valve, and which accumulated above it. By this expedient I did not hope to cure the disease, but only to empty the bowels, and to render the remnant of life supportable for the time she did live; at least it seemed to me to be giving the only possible chance of living, as my patient had not evacuated the alvine canal in nine weeks!"

Dr. De Leon was over-ruled—the patient began to sink from accumulated sufferings—vomiting was almost incessant—stercoraceous matters were ejected by the mouth—labour pains came on, and she was delivered of a well-formed fœtus of the sixth or seventh month, recently dead. In a few hours after parturition this unhappy lady paid the last debt of Nature.

*Dissection*, in the presence of Dr. Harris, and several medical students.*

"The body, when stript of its clothing, exhibited a spectacle uncommon in a woman of low stature and delicate frame.

* "Had I seen a case of 'stricture of the rectum,' reported by Dr. Jameson, of Baltimore, (before I had my last consultation,) in which he operated with success, I should certainly have performed the operation in my case. It will be recollected that I commenced the treatment of this patient on the 2d of March, and she died on the 16th of April. One month prior to the 2d of March she had not one alvine discharge, making the period of total obstruction two months and fourteen days!"

Its circumference, carefully measured from the crista of one os ilium to the opposite, was three feet and seven inches.

An incision was next made from the middle of the sternum to the pubes, and dissecting back the common covering, the peritoneum and omentum were exposed. These membranes were highly vascular, thickened, and inflamed, the vessels exhibiting a kind of net-work. The dissection was at this stage interrupted by the great effusion of fæces into the cavity of the abdomen. Whether this rupture of the intestine happened before or after death, it is impossible to determine: it did not, however, occur during the dissection. After securing the ruptured intestine, which we traced to be the ilium, we proceeded carefully with the examination, although a very disgusting task. The whole track of the intestines furnished an appearance and size I could not have believed possible in a human being! The circumference of the colon measured thirteen inches and a half. The duodenum was of equal size, the ilium not less than either. The whole canal was filled with fæces of the consistence of thick gruel, with a due admixture of bile. On the first view of the bowels I could assimilate their appearance to those only of the largest of the inferior animals. The whole mass was glued together, and the adhesions so very strict as not to be separated with the hand alone.

The intestines were now removed for further inspection, and after being emptied and washed, were examined. They exhibited a high degree of inflammation throughout their whole extent: they had lost their valvular structure, the canal being continuous. The fæces emptied out of the tube could not be measured, but the bystanders estimated the quantity at not less than from five to seven gallons. And from the enormous distention of the intestines it seemed not exaggerated.

Many days before her death, the exhalations from her body became so very offensive as to render her room insupportable to the attendants, in spite of every precaution that could be used to render it comfortable.

The *stomach* was much contracted, insomuch that at first sight it was overlooked; its coats had undergone an entire change; it was highly inflamed and thickened, containing feculent matter.

The *liver* was firm, but somewhat increased in size, otherwise healthy.

The *gall-bladder* sound and natural.

The *kidnies* had undergone no change whatever.

The *uterus* was minutely examined. It was very firmly contracted above the pubes, and resembled a solid body; the omentum adhered to it intimately. After dissecting it out, and cutting through its parietes, it opposed the knife with a crepitus not unlike the cutting through gristle; this scirrhosity pervaded its whole structure. The portion on which the placenta had been implanted, could scarcely be distinguished. The organ had little the appearance of so recently giving an outlet to so large a body as a child between the sixth and seventh months.

The *bladder* was small, contracted, and much changed from its structure and natural appearance.

The *rectum* was then examined, first carefully dissecting it out. Its whole structure had undergone a change; for the space of seven or eight inches it resembled gristle in texture and appearance. In many parts it approached nearer to the substance of bone, as it resisted the edge of the knife. As it reached within two or three inches of the sphincter ani, the canal was contracted to the size of a finger ring, resembling a circle of bone, and not admitting the little finger to pass entirely through it with all the force that I could exert. Through this ring the adventitious membrane escaped.

Before I dissected out the rectum, I introduced a large metallic bougie from above downwards, so as to verify my views as to the membrane spoken of in the history of the case. It was carried before the instrument without the sphincter ani. I investigated this pouch minutely, and could discover no opening in it by which the fæces could have escaped. During the lifetime of the patient I could always reach it with my finger introduced into the bowel, being pushed down in the form of a valve from the accumulation above it."*

---

* American Journal of the Medical Sciences, No. IV. August, 1828.

Well knowing the difficulties which beset the practitioner—the prejudices he has to encounter—and the necessity which he is often under, of balancing between a conscientious discharge of his duty and the risk of unmerited obloquy for so discharging it, we are not apt to examine, with a very critical eye, the judgment which is formed before the event has removed all doubt. Yet, in the foregoing case, we cannot see how doubt could exist as to the propriety of opening a passage that had been closed for so many weeks, and on the opening of which, the only chance of life remained. The fold of membrane acted completely as a valve in blocking up a scirrho-contracted rectum, and the puncture of this obstruction presented neither difficulty nor danger that ought, for a moment, to have weighed in the balance against the dreadful sufferings and miserable end of this unfortunate female! We know a gentleman in this metropolis, who is afflicted with an obstruction of this kind; but, fortunately he is able, with his own finger, to push up the valvular fold of relaxed mucous membrane, force it on one side, and thus permit the fæces to pass. We hope the foregoing case and dissection will be the means of saving some unhappy wretch from the horrors of a death such as is depicted by our transatlantic cotemporary.

---

